# A Patient Experience Course Syllabus: Integrating Service Sciences Research to Enhance Health-Care Delivery

**DOI:** 10.1177/2374373519870008

**Published:** 2019-08-21

**Authors:** Priyanka D Joshi, Stowe Shoemaker, Corrin C Sullivan, Neelesh R Soman

**Affiliations:** 1 College of Business, San Francisco State University, San Francisco, CA, USA; 2 Harrah’s Hospitality College, 14722 University of Nevada, Las Vegas, NV, USA; 3 School of Medicine, 14722 University of Nevada, Las Vegas, NV, USA; 4 Mountain View Hospital, Las Vegas, NV, USA

**Keywords:** patient experience, hospitality, service excellence, curriculum development

## Abstract

We present here a syllabus for teaching patient experience that draws on service sciences to address the current state of patient experience. The syllabus was the result of an ongoing collaboration between educators at the Hotel College and the School of Medicine at the University of Nevada at Las Vegas. The syllabus was developed after a thorough literature review in the field of services marketing, patient experience, hospitality marketing, management and leadership, health-care administration, and health-care communication and after consultation with subject matter experts. We believe that the syllabus provides an action plan for universities and hospitals to introduce and teach the topic of hospitality and patient experience as part of the medical and nursing school curriculum. The syllabus can also be adapted for teaching in executive education programs.

The leadership team at our medical school demonstrated a strong commitment to develop a medical education program with forefront in service provisioning and patient experience. With the goal to train physicians who were committed to providing excellent service to their patients, faculty at the school of medicine collaborated with experts in hospitality in the Hotel College in order to launch a hospitality in medicine program and train students about service. We realized that there was no preexisting syllabus or curriculum that we could adapt to train our students about hospitality. Given the lack of such an initial framework, we decided to engage in the groundwork needed to truly incorporate service sciences in our medical education.

We began our syllabus development efforts by doing a literature review of the current research on patient experience, health-care service delivery, and physician communication. We identified the most prevalent problems in service delivery in healthcare that were identified in this literature. We also gained a deeper understanding of the recommendations for enhancing patient experience that were provided by health-care professionals (1–7). We then drew on the vast research on hospitality management and services marketing as well as medical tourism (8) and health-care communication (9,10) to design an integrated syllabus that takes into account the most common patient experience and service delivery challenges faced by health-care professionals. The syllabus can be adapted for use with students and health-care professionals. In contrast to some of the previous work on medical tourism and concierge medicine (7,8) that emphasizes specific steps that an organization can take to enhance patient experience, our curriculum focuses on the processes that should guide patient experience decision-making by physicians and health-care workers.

After developing an initial syllabus, we consulted with 13 subject matter experts to obtain feedback on content, delivery methods, assignments, and ease of integration with medical curriculum. The subject matter experts included experts in medical curriculum and practicing and teaching physicians. We then revised the curriculum. Using this revised curriculum, we introduced the first 6 sections of our curriculum in 2 separate workshops conducted with our first-year medical students. Twenty-nine of the 60 students who attended our workshop completed the post-session evaluation questionnaire. Along with items about attitudes toward service, they completed 2 items about the hospitality sessions that are relevant here. Students were asked to indicate their agreement with the items: “The sessions on hospitality and communication covered relevant material” and “The instructor of the sessions on hospitality and communication was effective” on a 7-point scale ranging from 1 (strongly agree) to 7 (strongly disagree). Overall, the sessions were evaluated positively (see Table 1). We further revised the syllabus content based on our experience with teaching medical students.

**Table 1.** Descriptive Statistics Showing Evaluation of Workshops on Hospitality and Healthcare.^a^

Item	Sample Size	Mean (SD)	Median	Range
The sessions on hospitality and communication covered relevant material	29	1.79 (1.01)	2	1-5
The instructor of the session on hospitality and communication was effective	29	1.76 (1.12)	1	1-6

Abbreviation: SD, standard deviation.

^a^ Scale range: 1 = strongly agree; 7 = strongly disagree.

The finalized syllabus (see Appendix A) introduces critical concepts, theories, and models from the fields of service marketing and management that would serve to enhance student’s understanding of the patient’s experience and provide specific guidelines for addressing patient experience-related decision-making. Below, we provide 2 examples of concepts and theories that we believe are important in teaching service sciences in health-care settings and provide our rationale for introducing these models. Our intention here is 2-fold (1) to give the reader an understanding of how a service sciences lens to designing a patient experience syllabus may differ from a medical model and (2) to provide an initial framework to educators who are motivated to teach hospitality in health-care contexts. We believe that our syllabus can be used to teach a stand-alone course on patient experience or can be integrated into an intersession, doctoring, or patient communication course. The syllabus can be adapted for teaching in nursing, medical, or dental schools; in health-care administration programs; or in business schools.

## Components of Service Product

Patient experience can be defined as “the sum of all interactions, shaped by an organization’s culture that influence patient perceptions across the continuum of care (11).” One of the foundational concepts in teaching students about patient experience is understanding the components of service. In order to teach this concept, students need to understand the differences between services and products and the role of the customer in cocreation of service products. “Service customers expect value from access to labor, skills, expertise, goods, facilities, networks, and systems in exchange for money, time, and effort (12).” Unlike product customers who are paying for goods, service customers are paying for different components of the service product. The 3 components of services are the core product, the supplementary services, and the delivery process (12). For instance, when purchasing a burger at a restaurant, customers are paying not only for the burger (core product) but also for the scheduling and beverage service (supplementary services) and the server (delivery process). Similarly in health-care settings, the patient is not only paying for the core product which is the expertise of the physician but also for supplementary services such as the scheduling and information services, billings and insurance services, counseling services, and referral services. Patients also pay for the delivery process or how different aspects of core product and supplementary services are delivered to them through communication tools adopted by their health-care providers.

Health-care providers can gain a competitive edge by not only focusing on the core product or the efficacy of their diagnosis and treatment but also by providing exceptional supplementary services. Understanding the importance of the core product and supplementary services in predicting patient outcomes in different health-care settings allows the provider to focus their energy on improving the most critical aspects of patient experience in the particular context. For instance, patients may care more about supplementary services when they routinely visit their general practitioner, in which case it is important for the physician to be aware of these supplementary services in their ability to provide care. On the other hand, if the physician is a specialist who the patient does not see on a routine basis or does not expect to have long-term contact with, the supplementary services may be less important to the patient than the core product. It is important for the physician to truly evaluate the importance of the core product and the supplementary service or delivery process in the particular context that they encounter.

## Risk and Uncertainty in Health-Care Service

Another important difference between services and products is that services are inherently more risky than products due to difficulty in evaluating their quality prior to purchase. Services and products can be categorized as search goods, experience goods, and credence goods depending on the ease of evaluating their quality prior to and post purchase (13,14). Search goods are easy to evaluate prior to purchase—you can judge their quality and value by trying them prior to purchase. Most products (eg, jewelry, clothing) fall into this category. Experience goods are harder to evaluate and their value can be determined after consuming or experiencing the product or service. For instance, a bottle of wine can be judged by how it tastes after you have purchased and consumed it. This creates uncertainty and risk for the consumer who has to rely on reputation and reviews to make a purchase decision. Credence goods are the hardest to evaluate and their value can never truly be known with certainty—most medical services fall in this category. For instance, the efficacy of an implanted medical device may only be determined years after the patient has used it.

We further use this concept to elaborate on the different kinds of risks (functional or unsatisfactory performance risk, physical or personal injury risk, psychological risk, temporal risk, financial risk, social risk, and sensory risk) (15,16) that the patient experiences in the health-care context, allowing physicians to reflect on how each of these risk characteristics may play a role in the patient’s experience at their particular health-care setting. The health-care provider can then address these context-specific risks. Clinic images, materials, and staff communication can also seek to mitigate the risks that the patient encounters at the clinic. For instance, if patients at a particular clinic experience temporal risk in terms of long wait times, the staff can mitigate uncertainty and provide patients with greater cognitive control by providing information about average wait times.

The risks that patients experience in health-care contexts can be further elaborated upon and explored when covering topics of service delivery (17,18). It is important for students to be cognizant of patient expectations and experienced risks at the various touchpoints of their health-care experience. Creating a blueprint of the service experience in health-care contexts then serves to teach students about ways to enhance patient care at each touchpoint of patient interaction with their health-care system. Such a framework allows students to adapt their understanding of patient experience to the unique context in which they find themselves. For instance, a student completing a rotation at a primary care clinic may observe different critical touchpoints of patient experience than a student completing a rotation at a local hospital. Our syllabus serves to provide a broad framework that health-care providers can draw on in order to guide their decision-making about enhancing the patient’s experience of care depending on their specific context.

## Teaching Hospitality in Healthcare

In order for the health-care systems to truly be suited for enhancing patient experience, it is critical that health-care leaders understand the importance of service. A first step to promoting such an understanding of the importance of service experience of patients is to train physicians about hospitality and service leadership. Our syllabus serves as a guiding step for medical schools who wish to integrate hospitality and service in their teaching.

Our experience of teaching service sciences to medical students showed us that students were acutely aware of the need for improving the patient experience in most health-care settings and were highly motivated to learn about service and patient-centered care. We believe that it is important to introduce these concepts of hospitality and service early in the medical student education when students tend to have strong positive attitudes about patient-centered care (19,20). As students are interacting with patients in medical school, we believe that a service and patient experience lens will allow them to think about the patient experience and draw inferences about their own experiences with patients and staff in health-care settings. Given that many health-care settings have not yet made the transition to a patient-centered culture, these settings might serve as a tool for medical students to evaluate the quality of patient experience rather than assuming that the ongoing approach to patient care is the right one.

## Conclusion

By drawing on the large body of research in services management and marketing, educators can help medical students and physicians understand the different elements of providing exceptional service. We believe that our syllabus can be used for training of medical students, nurses, residents, physicians, and health-care administrators and leaders. We have also provided some recommendations for assignments, exercises, and cases that could be assigned to students in order to give them an opportunity to apply their learning to real-world experiences and contexts.

## Appendix A

### Patient Experience Course Syllabus

#### Course introduction

- Definition of patient experience
- Outcomes of patient experience—for the patient, for the employees, and for the organization

#### Creating value by understanding the service components of healthcare

- Definition of service
- Components of service experience (experience of core products vs supplementary services)
- Providing competitive edge through supplementary services

#### Empowering patients by enhancing perceived control

- The nature of risk in services versus products
- The high levels of inherent risks in health-care services and difficulty in evaluating quality
- Reducing the perceptions of risk in health-care contexts
- Enhancing patient’s ability to experience control (decision control, cognitive control, and predictive control) during their most vulnerable experiences

#### Understanding service expectations of patients: expectancy disconfirmation model

- Understanding patient expectations and zone of tolerance (the GAP model of service quality) at different stages of the health-care experience
- Service encounters as moments of truth—creating a blueprint of the service experience
- Service process redesign: Enhancing service at every touchpoint by exceeding expectations and mitigating risks.
- Measuring service quality

#### Building patient loyalty and trust

- Loyalty marketing versus frequency marketing
- Determining the lifetime value of a customer: The financial impact of word of mouth—both positive and negative word of mouth
- Introduction to the components of *The Loyalty Circle* (process, communication, and value), a framework for creating loyalty
- Strategies for enhancing patient loyalty

#### Leveraging communication to provide excellent service

- Communicating with patients: the role of empathy and patient voice (patients as cocreators of experience)
- Patient education and patient persuasion (strategies that work)
- Integrated service marketing communication
- Leveraging social media

#### Leading for service excellence

- Creating a culture of service orientation
- Hiring, training, and rewarding for service
- Empowering frontline employees
- Building high-performance service delivery teams
- Preventing burnout

#### Complaint handling and service recovery

- Understanding customer responses to service failures
- Customer expectations following complaints
- Principles of effective service recovery systems
- Discouraging abuse and opportunistic customer behavior

### Other Course Materials

#### Sample case studies

- Shouldice hospital—targeting service markets
- Massachusetts general—reengineering the service delivery process
- Frankenmuth case—word of mouth

#### Sample assignments

- Think about the last time you visited a health-care provider as a patient. What were the different elements of the service you received? Differentiate the quality of the core product from those of the supplementary services. Identify which aspects of the service was most important to you before you went in to see the doctor and which ones were most likely to influence your experience of care.
- What are patients expecting from their doctors? Understand patient expectations by talking to 5 patients.
- Observe the communication styles of 2 doctors. What are the similarities and differences in their communication practices? What are the persuasion strategies that doctors often use? When are they successful?
- Obtain feedback from a peer and a member of the staff about the quality of your service. Find out how the patient you spoke to today experienced your care (include a short instant patient input card).
- Read patient reviews on yelp: What are the common themes that emerge? What are the touchpoints in their clinic experience that patients have service complaints about? Are most service complaints related to core product or supplementary aspects of the experience?

#### Sample exercises

- Keeping a journal: Put yourself in the shoes of a patient you met today? How would the patient evaluate her experience? What were some strengths and weaknesses of the service the patient received?
- Encourage your patient to ask at least 3 questions? What do you notice about the questions they asked? How does this affect your relationship with your patient? (experimenting with patient experience)
- Change one thing about your patient care that is directed toward enhancing your patient’s experience of care or building trust and loyalty. (This exercise will allow us to introduce the stages of patient communication and the potential to enhance the patient’s experience at each stage: greeting, remembering physician name, give patients control, body position and questioning, the first question, communication plan, addressing patient discomfort, physician accent, and engendering confidence and trust in coworkers)
